# Treatment of renal cell carcinoma with a level III or level IV inferior vena cava thrombus using cardiopulmonary bypass and deep hypothermic circulatory arrest

**DOI:** 10.1186/s12957-015-0584-8

**Published:** 2015-04-22

**Authors:** Yong-Hui Chen, Xiao-Rong Wu, Zhen-Lei Hu, Wei-Jun Wang, Chen Jiang, Wen Kong, Wei Chen, Wei Xue, Dong-Ming Liu, Yi-Ran Huang

**Affiliations:** Department of Urology, Ren Ji Hospital, School of Medicine, Shanghai Jiao Tong University, 1630 Dongfang Road, Pudong District, Shanghai, 200127 China; Department of Cardiovascular Surgery, Ren Ji Hospital, School of Medicine, Shanghai Jiao Tong University, 1630 Dongfang Road, Pudong District, Shanghai, 200127 China

**Keywords:** Cardiopulmonary bypass, Hypothermic arrest, Renal cell carcinoma, Thrombectomy, Tumor thrombus

## Abstract

**Background:**

The aim of this study was to investigate the minimally invasive cardiopulmonary bypass (CPB) and deep hypothermic circulatory arrest (DHCA) approach in the management of renal cell carcinoma (RCC) with level III or IV inferior vena cava (IVC) thrombus and evaluate the survival outcomes.

**Methods:**

We performed a retrospective analysis on 32 RCC patients with IVC thrombus that underwent nephrectomy and thrombectomy via the minimally invasive CPB/DHCA approach between January 2007 and December 2013. Perioperative variables (for example, operative time, CPB duration, and circulatory arrest duration), estimated blood loss, hospital stay, perioperative complications, and survival data were recorded and analyzed.

**Results:**

Thirty-two patients (median age: 56 years) were treated surgically using the CPB and DHCA approach for RCC with a level III (*n* = 25) or level IV (*n* = 7) tumor thrombus. The median operation time was 360 min (interquartile range (IQR): 300 to 435 min) with median CPB and DHCA durations of 149 min and 23 min, respectively. The median estimated blood loss was 2,500 ml. Four complications were observed but no deaths occurred perioperatively. The median follow-up was 25 months (range: 4 to 64 months). The mean overall survival (OS) was 28.2 ± 4.6 months while the disease-free survival (DFS) was 19.5 ± 11.6 months. In patients with M0 disease, ten patients developed metastases and were treated with sorafenib as an adjuvant therapy. The mean OS and DFS of this subgroup were 25.4 ± 12.8 months and 16.0 ± 14.2 months, respectively.

**Conclusions:**

Radical nephrectomy and thrombectomy using CPB and DHCA to treat RCC is a relatively safe approach associated with low morbidity and mortality. This minimally invasive procedure may help minimize surgical trauma and improve perioperative outcomes.

## Background

Renal cell carcinoma (RCC), one of the most common forms of genitourinary malignancy, accounts for 3% of all solid neoplasms [1]. Due to an increase in high-resolution imaging, the incidence of RCC detection has increased. In approximately 20% to 30% of RCC cases, the patients either present with metastasis at the time of diagnosis or develop metastases after surgery [2]. Moreover, 4% to 10% of RCC patients also have a thrombus involving the inferior vena cava (IVC), and the tumor thrombus extends into the right atrium in 1% of these patients [3,4].

The intravascular growth observed in RCC patients could signify a heightened or more aggressive biologic behavior of the tumor. However, some authors agree that the RCC-associated tumor thrombus does not translate to a specific prognostic significance if it can be treated successfully with surgery [5]. To manage cases of RCC with IVC thrombus, several different surgical approaches such as liver transplantation [6,7] and thrombectomy using cardiopulmonary bypass (CPB) and deep hypothermic circulatory arrest (DHCA) [8,9] have been described. New developments in imaging, anesthesiology, and perioperative care have allowed for most thrombi to be removed successfully. There is, however, still considerable morbidity and mortality associated with treatment of this disease [10].

In the present study, we have retrospectively reviewed our management of RCC cases with level III and IV thrombi in a Chinese population using the minimally invasive CPB with DHCA approach. To the best of our knowledge, this study represents the largest analysis of surgical outcome in a Chinese population following this particular approach.

## Methods

### Patients

After Institutional Ethics Committee’s approval, we retrospectively reviewed all level III and IV tumor thrombus RCC cases operated on in the Urology Department of Ren Ji Hospital at Shanghai Jiao Tong University from January 2007 to December 2013. Tumor thrombus staging was performed according to the guidelines established by Neves and Zincke [11]. In total, 32 patients were included in this study. All patients were preoperatively evaluated with ultrasound, computed tomography (CT), or magnetic resonance imaging (MRI). To evaluate metastases, additional imaging modalities such as chest radiography, brain and lung CTs, and bone scans or PET-CT were employed if necessary. Clinicopathological features, including gender, age, which kidney was involved, tumor size, histological type, and tumor grade were obtained and analyzed. The total operation time, duration of the DHCA, estimated blood loss, number of transfusions and complications were also evaluated.

**Figure 1:**
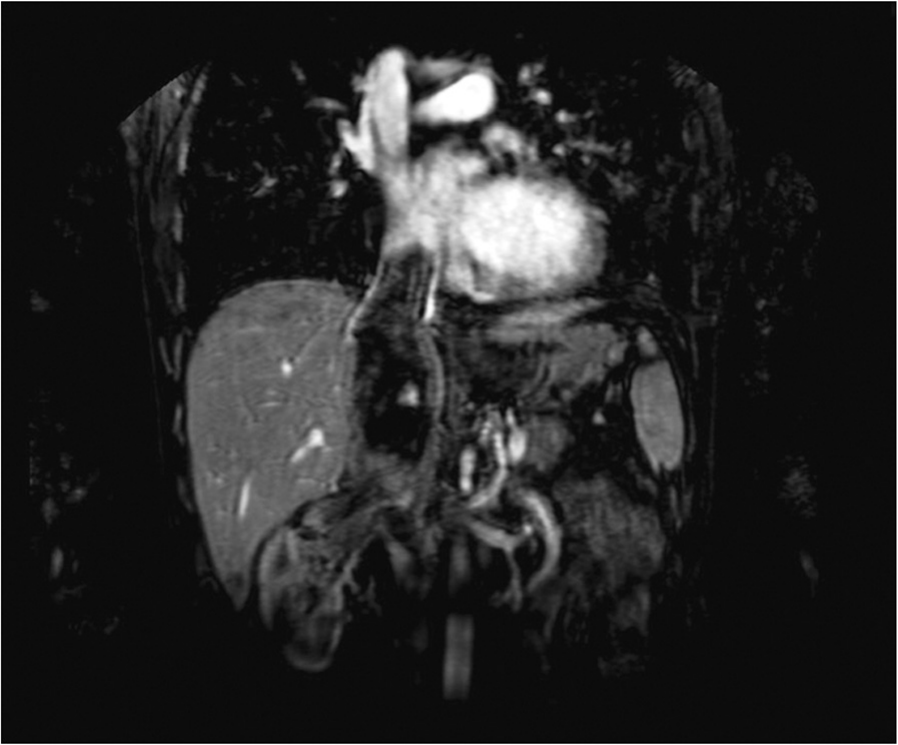
MRI of a right-sided renal cell carcinoma with right atrial tumor thrombus.

One patient in the case series, a 51-year-old man, presented with right kidney RCC and an IVC thrombus extending to the level of the diaphragm. Magnetic resonance angiography accurately depicted the cranial extension of the neoplastic thrombus (Figure 1). Results of lung CT and bone scan were negative. The patient has given consent for the report to be published.

### Operative technique

**Figure 2:**
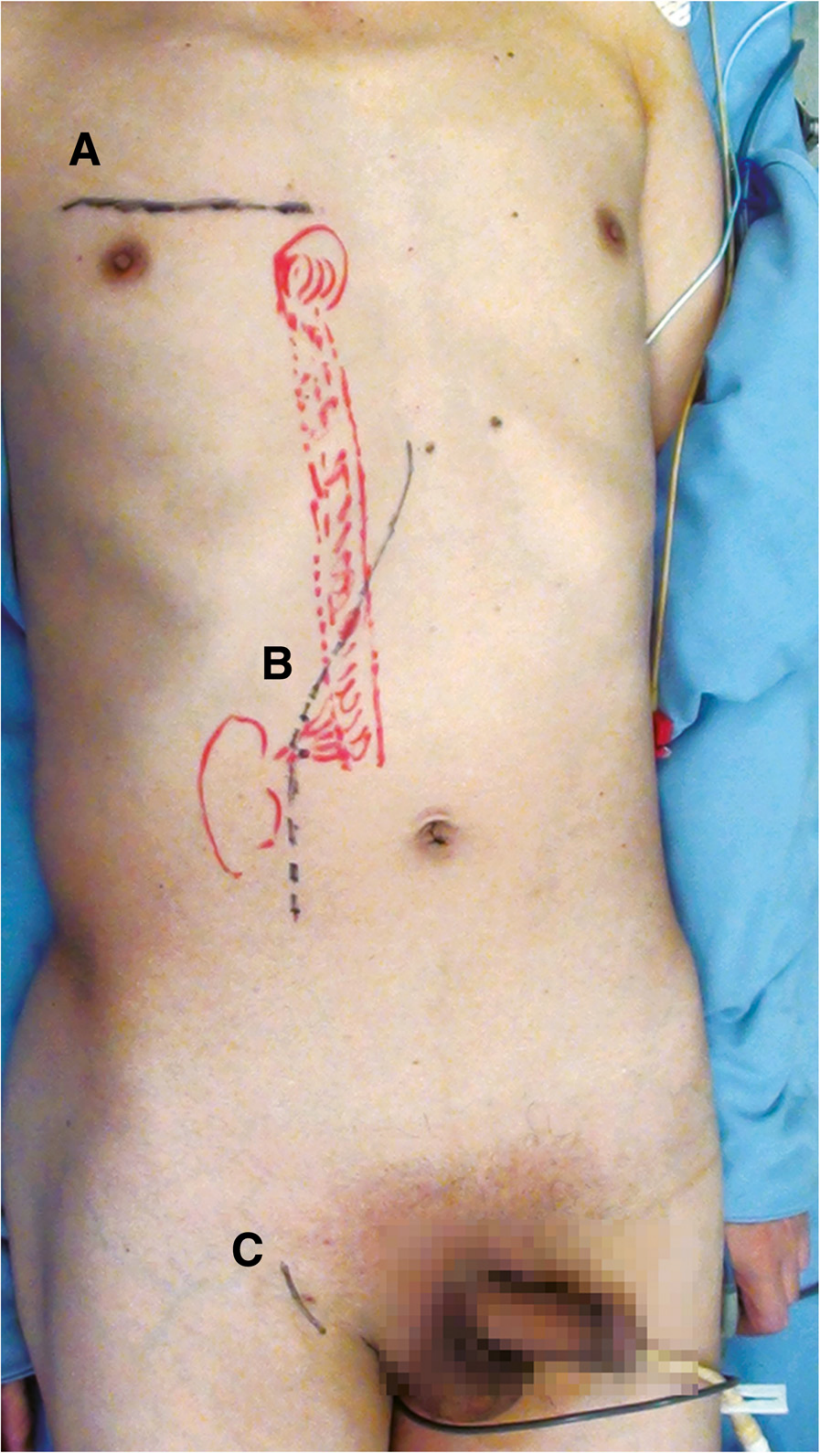
Incisions used for a right-sided tumor. An incision in the fourth intercostal space **(A)** and the right groin **(C)** were made to cannulate the superior vena cava and femoral artery. A subcostal incision alongside the abdominal rectus incision was made to expose the right renal and inferior vena cava **(B)**.

After the patient was placed in a supine position, a pararectal incision alongside the abdominal rectus incision (left: chevron incision) was made (Figure 2) and the abdomen was examined for evidence of tumor metastasis. The IVC and kidney were exposed, and the IVC was subsequently mobilized at the anterior surface only. An incision in the right groin was then made to expose the right femoral artery, and a minithoracotomy was performed in the fourth intercostal space to open the pericardium. Pericardial stay sutures were then passed through and stabilized outside the chest. The patient was systemically heparinized. The femoral artery and the right atrium were cannulated. The patient was placed on CPB and cooled to 18°C. DHCA was performed according to the protocol described by Welz *et al*. [12].

**Figure 3:**
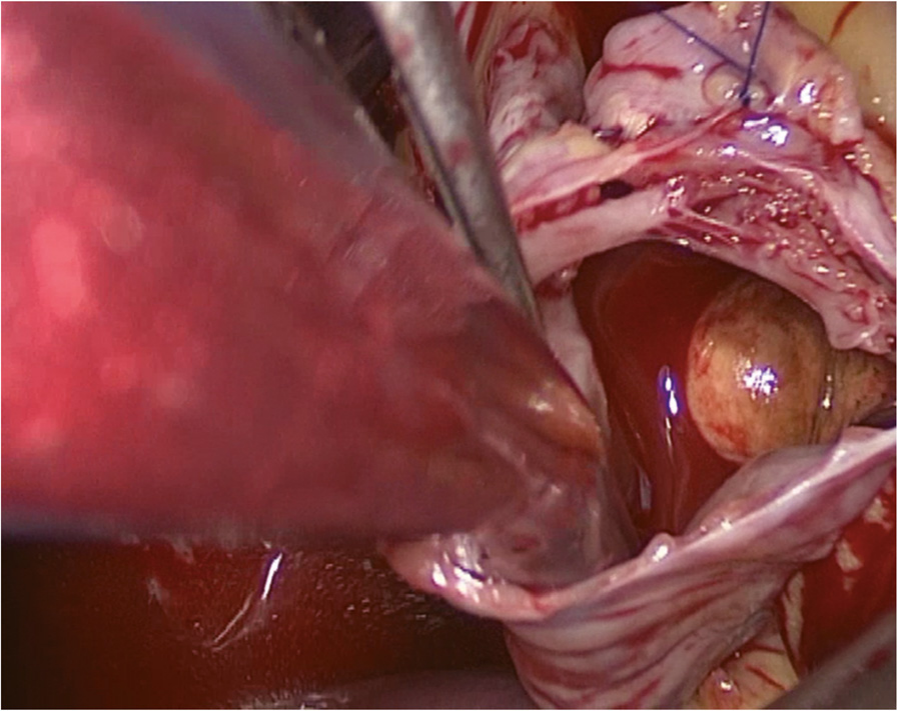
Atriotomy revealing a large atrial tumor thrombus.

**Figure 4:**
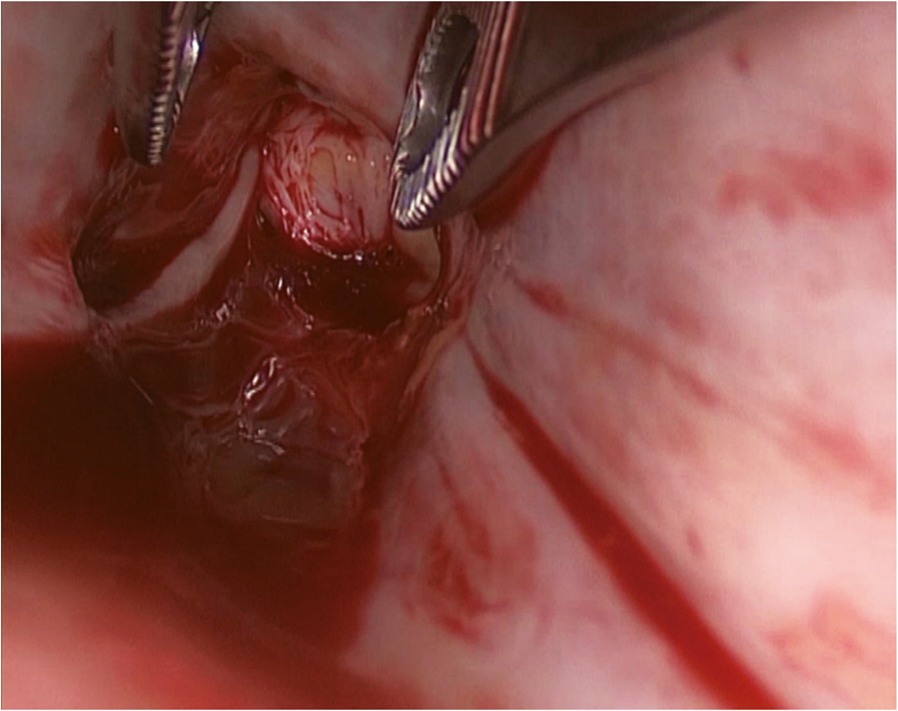
Laparoscopy was used to confirm the complete removal of the thrombus and determine whether the tumor was adherent to the IVC wall.

The IVC was mobilized as close to the diaphragm as possible, and the cephalad extension and the mobility of the tumor thrombus were confirmed using laparosonography. The venous collaterals, and the lumbar veins in particular, were ligated and sectioned, as hemorrhaging from these enlarged vessels is difficult to manage. After ligation of the lumbar veins, an anterior cavatomy was made to enable complete thrombus extraction. If necessary, an atriotomy was performed to gently milk the thrombus through the atriotomy into the IVC (Figure 3). After the thrombus was extracted from the IVC, laparoscopy or flexible cystoscopy was used to confirm complete tumor removal and whether the tumor was adherent to the IVC wall (Figure 4). If adherence was observed, a resection of the affected IVC segment was also performed.

**Figure 5:**
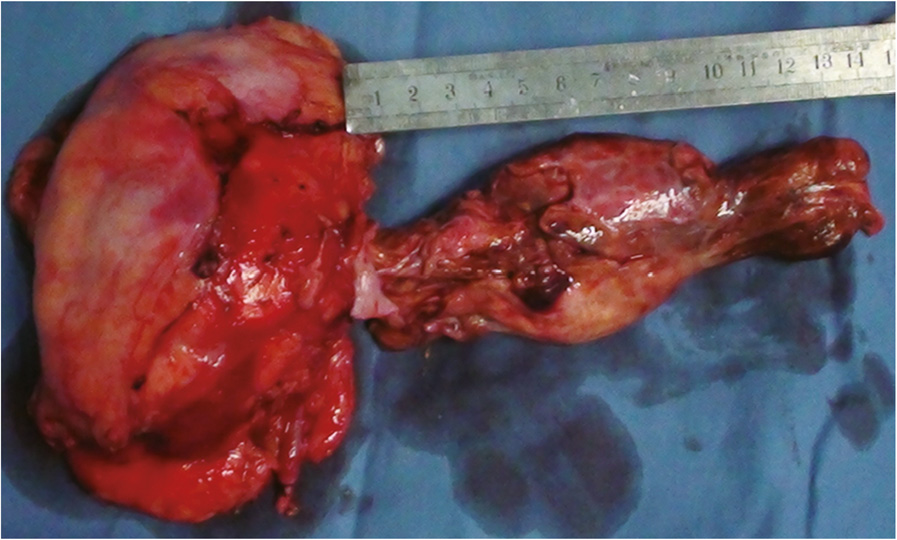
The specimen and tumor thrombus of a renal cell carcinoma patient with right atrial tumor thrombus invasion.

Prior to closure, a small IVC cuff at the renal vein junction was also taken. The right atrium and IVC were closed with 5-0 Prolene running sutures. After caval reconstruction and closure of the right atrium, the CPB was reestablished and the patient was rewarmed to 36°C. During this procedure, a radical nephrectomy was also performed (Figure 5).

## Results

Between January 2007 to December 2013, 32 consecutive RCC cases (19 men, 13 women) with a level III (*n* = 25) or level IV (*n* = 7) tumor thrombus underwent thrombectomy via the minimally invasive CPB and DHCA approach (Table 1). At the time of surgery, the median age was 56 years (IQR: 48 to 62 years). In 12 (37.5%) patients, the tumor was located on the left while 20 (62.5%) patients had a right-sided tumor.

**Table 1:** **Baseline characteristics of the patients**

**Parameters**	**Values**
Number of patients, *n*	32
Median age (IQR), years	56 (48 to 62)
Male/female, *n*	19/13
Left/right kidney, *n*	12/20
Thrombus level III/IV, *n*	25/7
Median tumor size (range), centimeters	12 (8 to 20)
Median (IQR) duration of	
Surgery, minutes	360 (300 to 435)
CPB, minutes	149 (138 to 177)
DHCA, minutes	23 (17 to 32)
Patients requiring IVC grafts, *n*	0
Median hospital stay (range), days	7 (5 to 10)
Estimated blood loss (IQR), milliliters	2,500 (1,650 to 4,000)
Tumor stage	
pT3b	24
pT3c	5
pT4	3
Tumor pathology	
Clear cell	25
Papillary	5
PENT	2

CPB, cardiopulmonary bypass; DHCA, deep hypothermic circulatory arrest; IQR, interquartile range; PENT, primitive neuroectodermal tumor.

The median total operation time was 360 min with median CPB and DHCA durations of 149 min and 23 min, respectively. The median estimated blood loss was 2,500 ml (IQR: 1,650 to 4,500), and 31 patients required a blood transfusion, with a median estimated blood transfusion volume of 2,600 ml (IQR: 1,000 to 4,400). The decision to administer blood was based on the degree of blood loss and on preoperative hemoglobin levels.

Four complications were observed in this patient cohort: acute renal failure (*n* = 1), liver dysfunction (*n* = 1), cardiac failure (*n* = 1), and coagulopathy (*n* = 1). We did not observe any cases of tumor embolism and no mortalities occurred due to surgical complications. Patients remained in the hospital for a median of 7 days (range: 5 to 10), and 1.5 days in the intensive care unit (range: 1 to 3). Pathologic examination of the tumors revealed that 25 (78.1%) patients had clear cell RCC, five (15.6%) patients had papillary RCC, and two (6.3%) patients had primitive neuroectodermal tumors (PENT). Three patients presented with tumor thrombi that invaded the IVC wall, such that part of the IVC luminal wall was resected. The longitudinal caval incision was closed with running sutures and none of the patients required IVC segment resection or IVC grafts. The median tumor size was 12 cm (range: 8 to 20).

The median follow-up for the patients was 25 months (range: 4 to 64). The mean overall survival (OS) period of the cohort was 28.2 ± 4.6 months, while the disease-free survival (DFS) period was 19.5 ± 11.6 months. Two patients (6.25%) in our population had M1 disease, which is lower than what has been reported in other previously published cohorts, and both of these patients died. The mean OS of the two M1 patients was 4.5 months. In the patients with M0 disease, ten (33.3%) developed metastases. All patients with metastases were administered sorafenib as an adjuvant therapy. The mean OS and DFS of this subgroup were 25.4 ± 12.8 months and 16.0 ± 14.2 months, respectively. The mean OS of patients without metastases was 33.0 ± 18.7 months.

## Discussion

Medical therapies have proven ineffective in the treatment of RCC with IVC thrombosis, and radical nephrectomy together with thrombectomy is the only effective therapeutic alternative for these patients [9,13]. Surgical management of RCC in which there is extension into the renal vein, IVC, or even the right atrium, however, poses a significant technical challenge [10]. With the technological advances in imaging, anesthesiology, and cardiosurgery, surgical treatment of RCC with an IVC thrombus is now feasible, and the thrombus can almost always be removed successfully.

Although the benefit of CPB and DHCA in RCC patients with level IV thrombus is obvious, controversy still persists in the treatment of RCC patients with a level III thrombus. Some experts suggest the use of non-extracorporeal circulation and hypothermic circulatory arrest to facilitate the removal of the tumor thrombus [6,7]. High level thrombi can also be managed with venovenous bypass via a caval-atrial shunt [14]. This technique avoids CPB and circulatory arrest, but it may result in hepatic venous bleeding even when performed by experienced surgeons. At our institution, we prefer an extracorporeal circulation approach, as the use of CPB and DHCA decrease the risk for unexpected and life-threatening intraoperative hemorrhage and incomplete tumor extirpation, which are the primary goals of the surgery. With help from the Department of Cardiac Surgery, CPB and DHCA have been used to treat RCC with level III or IV IVC thrombi in our hospital since 1997.

We have made some technical modifications to this surgical approach in comparison to the published procedure [12]. First, after a pararectal (for right RCC) or chevron incision (for left RCC) was made and the metastases were assessed, only the anterior part of the IVC was mobilized to reduce kidney mobilization to prevent tumor embolism. Second, none of the patients in this cohort underwent preoperative renal artery embolization, as it is associated with increased complications and perioperative mortality [13]. Third, a minithoracotomy was performed in the fourth intercostal space in lieu of a median sternotomy to open the pericardium. A minithoracotomy allows for a rapid postoperative recovery and reduces the use of analgesics and the length of hospital stay [15]. As previously reported [8], because the minithoracotomy has only a small incision, there are esthetic advantages. This technique is faster and has fewer requirements for mechanical ventilation and transfusion than traditional median sternotomy [8,9].

In this patient cohort, after CPB was established and during the procedure of cooling the body to a core temperature of 18°C, the IVC was mobilized. Once DHCA was achieved, the thrombectomy was performed. After vascular reconstruction was complete, the patient was rewarmed. During the rewarming period, a radical nephrectomy and lymphadenectomy were performed. Performing the nephrectomy and lymphadenectomy during the rewarming period reduces the waiting time during the cardiosurgical portion of the procedure and shortens the total operation time.

IVC thrombus is often associated with tumor metastasis. Most of the previously published cohorts report that 30% of patients have M1 disease [3], and IVC thrombectomy is advocated as it may relieve the symptoms and potentially allow for a better quality of life [3,10]. Postoperative targeted adjuvant therapy with either sorafenib or sunitinib in patients with clear cell RCC, who are at a high risk for disease recurrence, and it is effective in reducing the recurrence rate [16]. This promising result warrants further analysis in randomized clinical trials with larger patient populations.

Our study has several limitations. It was performed retrospectively in a single institution, and there was some bias in patient selection. The study population was small and the number of patients with M1 disease was lower than other published series [3,8]. Future studies should be conducted to compare this technique with other surgical techniques for the management of RCC with a level III or IV tumor thrombus.

## Conclusions

Patients with level III or IV IVC thrombus RCC can be treated safely and effectively via radical nephrectomy and thrombectomy using CPB combined with DHCA. This approach is associated with low rates of morbidity and mortality. Introduction of a right anterior minithoracotomy may minimize surgical trauma, accelerate postoperative recovery, and improve perioperative outcomes.

## Abbreviations

CPB: cardiopulmonary bypass
DHCA: deep hypothermic circulatory arrest
IVC: inferior vena cava
PENT: primitive neuroectodermal tumors
RCC: renal cell carcinoma
